# A comprehensive functional analysis of tissue specificity of human gene expression

**DOI:** 10.1186/1741-7007-6-49

**Published:** 2008-11-12

**Authors:** Zoltán Dezső, Yuri Nikolsky, Evgeny Sviridov, Weiwei Shi, Tatiana Serebriyskaya, Damir Dosymbekov, Andrej Bugrim, Eugene Rakhmatulin, Richard J Brennan, Alexey Guryanov, Kelly Li, Julie Blake, Raymond R Samaha, Tatiana Nikolskaya

**Affiliations:** 1 GeneGo, Inc. Renaissance Drive, St. Joseph, MI 49085, USA; 2 Vavilov Institute of General Genetics, Russian Academy of Sciences, Gubkina Str, Moscow, Russia; 3 Applied Biosystems, Inc, Lincoln Center Drive, Foster City, CA 94404, USA

## Abstract

**Background:**

In recent years, the maturation of microarray technology has allowed the genome-wide analysis of gene expression patterns to identify tissue-specific and ubiquitously expressed ('housekeeping') genes. We have performed a functional and topological analysis of housekeeping and tissue-specific networks to identify universally necessary biological processes, and those unique to or characteristic of particular tissues.

**Results:**

We measured whole genome expression in 31 human tissues, identifying 2374 housekeeping genes expressed in all tissues, and genes uniquely expressed in each tissue. Comprehensive functional analysis showed that the housekeeping set is substantially larger than previously thought, and is enriched with vital processes such as oxidative phosphorylation, ubiquitin-dependent proteolysis, translation and energy metabolism. Network topology of the housekeeping network was characterized by higher connectivity and shorter paths between the proteins than the global network. Ontology enrichment scoring and network topology of tissue-specific genes were consistent with each tissue's function and expression patterns clustered together in accordance with tissue origin. Tissue-specific genes were twice as likely as housekeeping genes to be drug targets, allowing the identification of tissue 'signature networks' that will facilitate the discovery of new therapeutic targets and biomarkers of tissue-targeted diseases.

**Conclusion:**

A comprehensive functional analysis of housekeeping and tissue-specific genes showed that the biological function of housekeeping and tissue-specific genes was consistent with tissue origin. Network analysis revealed that tissue-specific networks have distinct network properties related to each tissue's function. Tissue 'signature networks' promise to be a rich source of targets and biomarkers for disease treatment and diagnosis.

## Background

The issue of tissue and cell-type specificity of gene expression is central to human biology and biomedicine. It impacts such fundamental problems as tissue ontogenesis, evolution and carcinogenesis. It is generally believed that the most relevant disease biomarkers and drug targets predominantly are found among proteins specific for the tissue the disease affects. In 1965 Watson et al. described genes universally expressed to maintain cellular functions as 'housekeeping' (HK) genes [1]. Comprehensive experimentation in this area was, however, limited until, in the 1990s, microarrays enabled 'genome-wide' snapshots of gene expression. Numerous studies on tissue-specific expression have since been published [2-8], identifying between 451 and 1789 genes [4] as a HK core on different microarray platforms. There is no standard method for assigning a gene as HK, and no comprehensive functional analysis of HK and tissue-specific genes has previously been done.

Here we report the identification of 2374 HK genes, based on a novel study of gene expression in 31 human tissues on a whole-genome ABI array. We set up a definitive 'HK baseline' for gene expression across tissues, and compared previously published HK data sets with ours. We also identified gene sets uniquely expressed in individual tissues and groups of tissues, and generated tissue-specific merged metabolic/signaling 'signature networks'. Both HK and tissue-specific genes were subjected to a comprehensive, three-phase functional analysis, including gene set enrichment across four ontologies, network topology analysis and tissue-specific network analysis. We clustered tissues into groups according to expression patterns and network parameters, and revealed associations between HK and tissue-specific genes with human diseases and drug targets.

## Results

### Definition of housekeeping and tissue-specific genes

**Figure 1:**
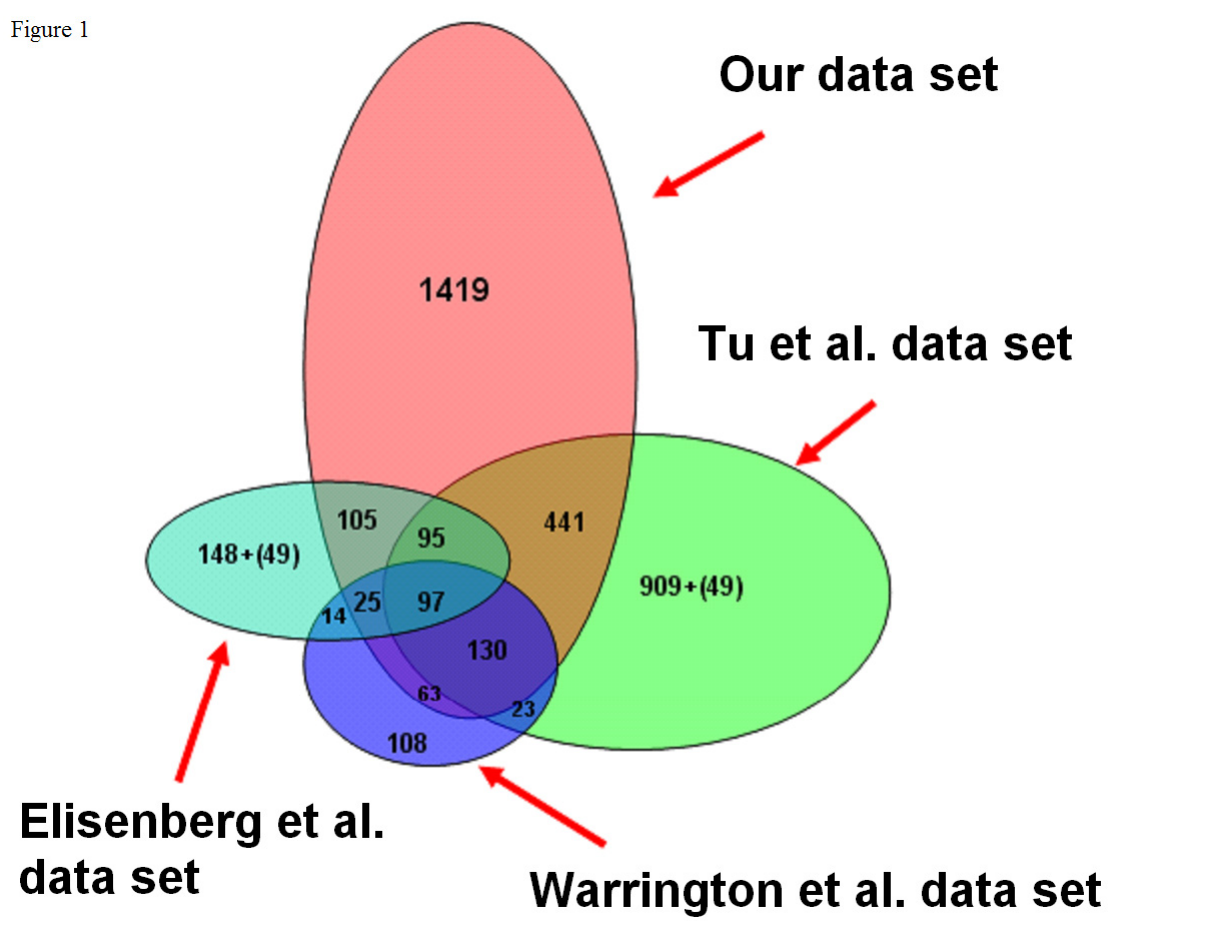
**Comparison between housekeeping gene sets**. The intersections between Tu et al. [4], Warrington et al. [3], Eisenberg and Levanon [2] and our set are shown. The smallest of the four previously published sets [5] was not included in the figure for clarity. The numbers in parentheses are the number of unique genes that overlap between the sets located at the opposite sides.

Whole-genome gene expression was analyzed in 31 human tissues using an ABI human genome array with probe sets for 27,868 individual human genes. A relatively conservative ten-fold 'signal-to-noise' ratio (SN10) across all three replicate arrays for each tissue was applied to identify the transcripts present in all tissues (HK set, see Methods). The SN10 HK set comprised 2374 genes (Additional file 1). We compared this set with four sets of HK genes published previously: a set of 535 genes from 11 tissues on a Affymetrix HuGeneFL array [3]; 1789 genes from 79 tissues on custom 33,698 gene array [4]; 574 genes from 47 tissues on a 7500 gene Affymtrix U95A array [2] and 451 genes from 19 tissues on Affymetrix HuGeneFL arrays [5]. Our HK set overlapped with between 42 and 82% of these sets, although only 97 genes were common between the four larger sets. Furthermore, there was 80% overlap between our set and the intersection of the previous studies (Figure 1). Our set contained 1419 additional HK genes not previously identified.

Genes uniquely expressed in only one of the 31 tissues (at the SN10 cut-off) were defined as tissue specific. Tissue-specific sets varied in size from four genes for thymus to 484 genes for testis, with an average size of 43.8 genes, or 30.9 genes for somatic tissues only (excluding testis) (Table 1, Additional file 2). The gene set uniquely expressed in testis was substantially larger than those for somatic tissues, and highly enriched in meiosis-specific genes, confirming recent findings [9,10]. Around 40% of a recently published human testis specific gene set [11] overlapped with our SN10 set.

**Table 1:** Number of housekeeping and tissue-specific genes

**Tissues**	**SN10**
Housekeeping	2374
Liver	22
Skeletal muscle	37
Fetal liver	16
Testis	484
Placenta	38
Bone marrow	63
Skin	75
Adrenal gland	13
Prostate	14
Trachea	16
Small intestine	35
Peripheral blood lymphocytes	49
Mammary gland	16
Tonsil	24
Thymus	4
Spleen	14
Fetal kidney	5
Thyroid	7
Brain	34
Heart	26
Lung	16
Salivary gland	17
Ovary	15
Pancreas	20
Fetal thymus	8
Colon	9
Spinal cord	24
Retina	190
Kidney	17
Uterus	12
Fetal brain	61
**Average**	43.8
**Average, somatic tissues**	30.9

An alternative method, using the Student's *t*-test, was also applied to identify tissue-specific genes. Functional analysis showed striking similarities in the biological processes encompassed in the genes specific to each tissue between the *t*-test and the threshold-based methods (description of method and results in Additional file 3).

### Ontological analysis of housekeeping and tissue-specific genes

**Figure 2:**
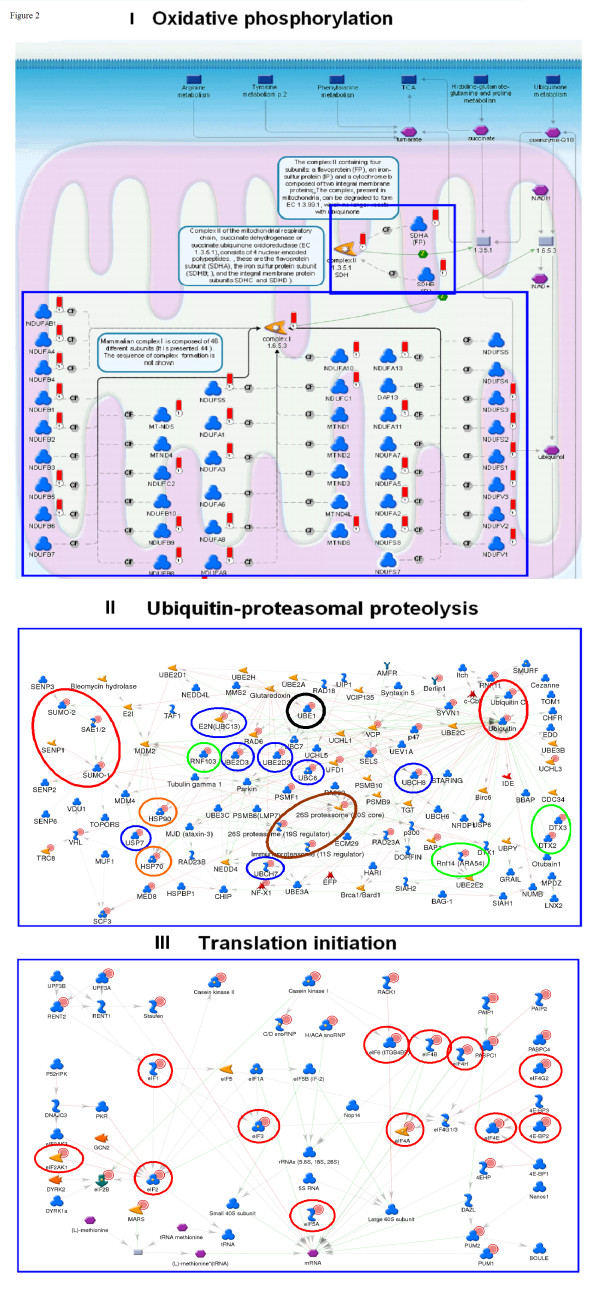
**Top-scored pathways maps and process networks for housekeeping proteins**. (I) Oxidative phosphorylation map. The subunits of the complexes are grouped in blue boxes; the red 'thermometer' histograms mark housekeeping (HK) genes. (II) Network for ubiquitin-mediated protein degradation in proteosome. HK genes are marked with solid red circles. The most important network components functionally, identified as HK genes, are marked with circles: ubiquitins and small ubiquitin-like modifiers (Ubiquitin, Ubiquitin C, Sumo-1, Sumo-2 are encircled in red); the ubiquitin-activating enzyme (UBE1 is encircled in black); ubiquitin-conjugating enzymes (UBCH8, UBE2D2, UBE2D3, UBC13, UBCH7 are encircled in blue); proteins that may act as ubiquitin protein ligases E3 (DTX2, DTX3, Rnf14, Rnf103 are encircled in green); the chaperons (HSP70 and HSP90 encircled in orange) and proteasomal subunits (26S proteasome, 26S proteasome, immunoproteasome (11S regulator) are encircled in brown). (III) Network for the GeneGo process translation initiation. HK genes are marked with solid red circles and translation initiation factors are encircled in red.

We conducted enrichment analysis of HK and tissue-specific sets across four functional ontologies: canonical pathway maps, gene ontology (GO) processes, GeneGo (GG) process networks, and diseases, using the hypergeometric distribution [12] and Gene Set Enrichment Analysis (GSEA) algorithms [13] to determine enrichment. The hypergeometric *p*-values for the ten top-scored entities in each ontology for the HK set are shown in Table 2 and Additional file 4. 'Oxidative phosphorylation' was the highest scored canonical pathway map (*p *< 10–77). This vital, ATP-yielding pathway is the terminal part of cellular respiration, in which electrons are transferred through the electron transport chain (ETC). Almost all subunits of the ETC protein complexes were expressed in the HK set (Figure 2(I)). The second-highest scored map depicts another essential process in the mitochondrial respiratory chain, ubiquinone metbolism (*p *< 10–40). Ubiquitin-proteasomal proteolysis was among the highest scored GG process networks (*p *< 10–26), with virtually all essential proteins included in the HK gene set (Figure 2(II)). GG process ontology is a set of 120 networks covering most critical cellular processes. Each GG process comprises 100 to 350 functionally related proteins. Unlike GO terms, where proteins are not necessarily connected, the proteins in GG processes are linked via physical interactions, and can be visualized as networks. They therefore represent the signal flow and metabolic flux within each process. Translation initiation was another high-scored GG process (Figure 2(III)), with many translation initiation factors represented in the HK set. In GO biological processes, cellular metabolism, translation and RNA processing were among the top ten scoring categories. Neoplasm by site (*p *< 10–16) and breast neoplasm (*p *< 10–14) were the top-scored diseases (Table 2). Additionally, enrichment analysis of canonical pathway maps showed that nine out of the top ten maps are directly connected to 'growth' and 'viability' (Additional file 5).

**Table 2:** Enrichment analysis of housekeeping and three selected tissue-specific gene sets.

	**Housekeeping**	**Retina**	**Testis**	**Adrenal Gland**
**Canonical pathways maps**	Oxidative phosphorylation	Visual perception	CREM signaling in testis	Tyrosine metabolism (dopamine)
	Ubiquinone metabolism	CREB pathway	Glycolysis and gluconeogenesis	Catecholamine metabolism
	Cytoskeleton remodeling	TGF, WNT and cytoskeletal remodeling	ATM/ATR regulation of G2/M checkpoint	Estrogen biosynthesis
	Formation of Sin3A and NuRD complexes and their role in transcription regulation	Regulation of CDK5 in CNS	Role of APC in cell cycle regulation	Cortisone biosynthesis and metabolism
	TGF, WNT and cytoskeleton remodeling	Role of Nek in cell cycle regulation	Rap1A regulation pathway	Androstenedione and testosterone biosynthesis and metabolism
	Regulation activity of EIF4F	Plasmin signaling	Androgen receptor nuclear signaling	Non-genomic (rapid) action of androgen receptor
	WNT signaling pathway	Receptor-mediated HIF regulation	Insulin regulation of fatty acid metabolism	Androgen receptor nuclear signaling
	Role of tetraspanins in the integrin-mediated cell adhesion	Sphingolipid metabolism	Rap1B regulation pathway	WNT signaling pathway
	BAD phosphorylation	Retinol metabolism	Nucleocytoplasmic transport of CDK/Cyclins	TGF, WNT and cytoskeletal remodeling
	TCA	Regulation activity of EIF2	Estrogen biosynthesis	
**GeneGo processes**	Translation_Translation initiation	Signal transduction_Visual perception	Reproduction_Spermatogenesis	Transport_Potassium transport
	Translation_Elongation-Termination	Transmission of nerve impulse	Cell cycle_Meiosis	Cell adhesion cadherins
	Transcription_mRNA processing	Reproduction_GnRH signaling pathways	Signal transduction_CREM pathway	Development neurogenesis in general
	Proteolysis_Ubiquitin-proteasomal proteolysis	Development_Neurogenesis in general	Reproduction_Male sex differentiation	Signal transduction NOTCH signaling
	Translation_Regulation of initiation	Transport_Calcium transport	Signal transduction_Androgen receptor nuclear signaling	Signal transduction_Androgen receptor signaling cross talk
	Protein folding_Folding in normal condition	Development_Neurogenesis- synaptogenesis	Cell cycle_Mitosis	Signal transduction_CREM pathway
	Immune_Phagosome in antigen presentation	Reproduction_Progesterone signaling	Proteolysis_Ubiquitin-proteasomal proteolysis	Signal transduction_Neuropeptides signaling pathway
	Protein folding_Protein folding nucleus	Cell adhesion_Integrin priming	Cell cycle_Core	Development ossification and bone remodeling
	Protein folding_Response to unfolded protein	Translation_Regulation of initiation	Signal transduction_Visual perception	Muscle contraction
	Protein folding_ER and cytoplasm	Reproduction_Male sex differentiation	Cell cycle_G2-M	Cell adhesion amyloid proteins
**GO processes**	Metabolic process	Sensory perception of light stimulus	Sexual reproduction	Feeding behavior
	Cellular metabolic process	Visual perception	Reproduction	Smooth muscle differentiation
	Translation	Sensory perception	Spermatogenesis	Oocyte development
	Macromolecule metabolic process	Neurological process	Male gamete generation	Oogenesis
	Primary metabolic process	Detection of visible light	Gametogenesis	Male gonad development
	Macromolecule biosynthetic process	Detection of light stimulus	Fertilization	Embryonic epithelial tube formation
	Cellular protein metabolic process	Detection of abiotic stimulus	Sperm motility	Development of primary male sexual characteristic
	Cellular macromolecule metabolic process	Phototransduction	Spermatid development	Germ cell development
	Protein metabolic process	Detection of light stimulus during visual perception	Spermatid differentiation	Cell-cell signaling
	Intracellular transport	Detection of light stimulus during sensory perception	Development of primary sexual characteristics	Dopamine biosynthetic process from tyrosine
**Diseases**	Neoplasm by site	Vision disorder	Infertility	Diabetes insipidus
	Breast neoplasms	Eye diseases	Infertility, male	Hypopituitarism
	Breast disease	Retinal degeneration	Dyskeratosis congenita	Adrenal gland disease
	Genetic disease, inborn	Sensation disorders	Dysautonomia, familial	Adrenal cortex disease
	Digestive systems neoplasm	Night blindness	Ciliary motility disorders	Adrenal cortex neoplasm
	Poxviridae infections	Retinitis pigmentosa	Kartagener Syndrome	Adrenal gland neoplasm
	Lysosomal storage disease	Eye diseases, hereditary	Dextrocardia	Carcinoma, basal cell
	Mental retardation	Retinal diseases	Herpes zoster	Neoplasm, basal cell
	Aneuploidy	Blindness	Bronchiectasis	Myokymia
	Vaccinia	Retinitis	Autonomic nervous system diseases	Pathologic processes

Gene set enrichment with canonical maps, GeneGo processes, gene ontology processes and diseases was performed.

Using the GSEA procedure [13] across the same ontologies produced similar distributions (data not shown). Importantly, HK sets from the previously published studies were generally enriched in similar categories to our own HK set across all four ontologies. For example, oxidative phosphorylation, cytoskeleton remodeling, translation and macromolecule biosynthetic process were among the top-scoring maps and processes for all HK sets (Additional file 4). Functional analysis of the unique parts of HK sets showed differences among top-scoring processes and maps. Our unique (not yet identified) HK genes showed enrichment with metabolic, translation and cell-cycle processes, validating the part of the HK set which was not identified by previous studies. Unique parts of some of the other sets, however, showed enrichment of more specific processes such as immune, inflammatory and cardiac-specific processes (Additional file 6).

**Figure 3:**
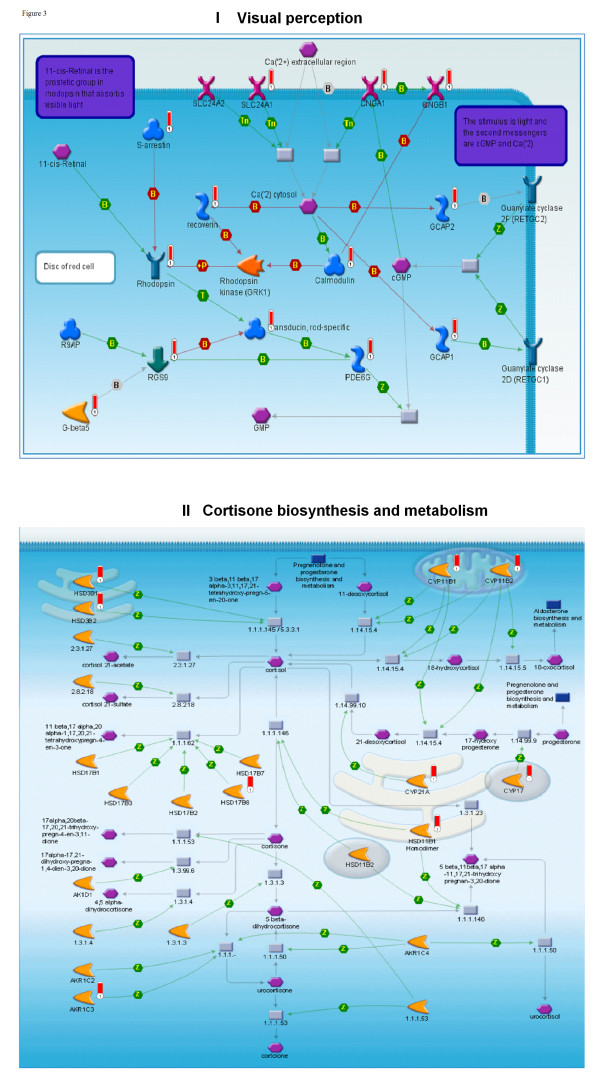
**Top-scored canonical maps for tissue-specific genesets**. (I) Retina, visual perception canonical map. The genes identified as expressed in retina are marked with red 'thermometer' icons. (II) Cortisone biosynthesis and metabolism canonical map. The genes identified as present in adrenal gland are marked with red 'thermometer' icons.

All 31 tissue-specific gene lists were subjected to the same enrichment procedures as the HK set (Additional file 7). In most cases, *p*-value distributions of top-scored pathways, processes and diseases were strikingly consistent with the tissue of origin. For instance, 190 retina-specific genes were enriched for eye-specific categories in all four ontologies (Table 2). Visual perception (Figure 3(I)) and retinoid metabolism, both highly specific for retina tissue, were among the top ten scored pathways maps. The top-scored GG process network was Signal transduction_Visual perception (Table 2). All ten top-scored GO processes were related to vision, including sensory perception of light stimuli, visual perception and detection of visible light. All ten top-scored diseases were eye diseases, including retinal degradation, night blindness and retinitis pigmentosa. Adrenal gland genes were enriched in disease categories including diabetes insipidus, adrenal gland diseases and adrenal cortex diseases (Table 2). The testis-specific set was highly enriched in cell cycle (meiosis), DNA exchange and cell division in all three process ontologies (canonical pathways, GO processes and GG processes). The top-scored diseases included male infertility, dyskeratosis congenita and familial dysautonomia (Table 2). Brain-specific genes yielded GO processes such as transmission of nerve impulse and synaptic transmission. Disease enrichment for the brain set included epilepsy, seizures and mental disorders (Additional file 7). Using the GSEA procedure [13] across the same ontologies produced similar distributions (Additional file 8).

### Network properties and interactome analysis of HK and tissue-specific genes

We analyzed the 'interaction space' of the protein products of HK and tissue-specific genes in three steps. First, we calculated topological properties of the 'interactomes', such as degree of connectivity, average shortest path and clustering coefficient [14]. Second, we evaluated the interactome structure by parsing the interactions into three components (IN, OUT and giant strong component, GSC) using the 'bow-tie' classification of Broder et al. [15]. Third, we divided HK and tissue proteins into protein classes and calculated relative enrichment across particular classes. For topological analysis, we used the directional protein-protein interaction content in MetaCore™ (GeneGo Inc.). We also analyzed, for reference, four previously published HK gene sets.

**Figure 4:**
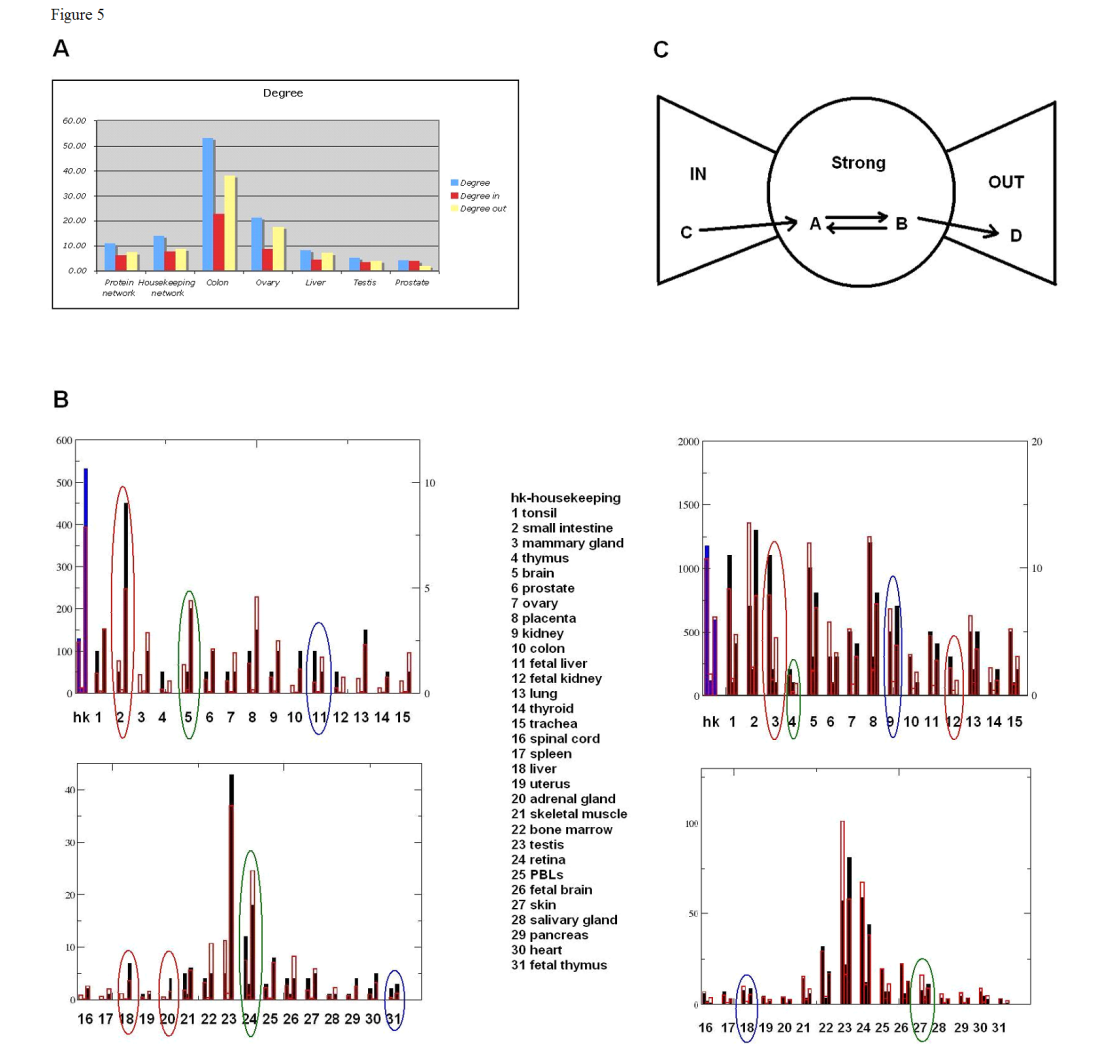
**Network analysis and protein class enrichment of housekeeping and tissue-specific genes**. A. Average connectivity of global protein interaction, housekeeping (HK) and several tissue-specific gene sets. B. Protein class enrichment analysis. The actual (black or blue bars) and expected (open red box) number of transcription factors (first bar), receptors (second bar) and enzymes (third bar) for each tissue. The blue bars correspond to HK proteins. The circles identify several tissues enriched in transcriptional factors (blue), receptors (green) and enzymes (red). C. Network component enrichment analysis. The upper part of the figure shows the schematic illustration of IN, OUT and GSC (figure adopted from Brodel et al. [15]). The lower panel shows the actual (black and blue bars) and expected (open red boxes) number of genes in the GSC (first), IN (second) and OUT (third) components. The blue bars correspond to housekeeping proteins. The circles identify several tissues enriched in GSC proteins (red), IN proteins (green) and OUT (blue).

All five HK sets shared similar network topology features: higher connectivity, a somewhat lower clustering coefficient, and shorter paths than the global human interactome (Figure 4A, Additional file 9). The tissue-specific networks varied substantially in their degree of connectivity, with colon and ovary-specific proteins the most interconnected, and prostate and salivary gland the least (Figure 4A, Additional file 10).

Network topology parameters were also used for tissue clustering. The topological distances between all possible tissue pairs were calculated as the ratio of the average shortest paths between proteins belonging to different tissues to the average shortest paths between proteins from the same tissue. Standard hierarchical clustering [16] was applied to generate the tissue tree. Interestingly, some of the functionally related tissues such as brain, fetal brain, retina and spinal cord were grouped together and characterized by very similar average shortest paths (Additional file 10).

An interactome can be divided into three structural parts, or 'components' [15]. The GSC is the most densely connected part of the network, characterized by the property that any two nodes can be connected through directed paths in both directions. Directed paths from the GSC lead out to the OUT component and paths from the IN component go in to the GSC (Figure 4C, upper panel). In order to facilitate interpretation, the interactions were divided by 16 mechanisms (Additional file 11). The IN component is enriched with 'outgoing' interactions such as ligand-receptor binding interactions, which occur five times more frequently in IN than in GSC, and ten times more frequently than in the OUT component. Interactions between IN and GSC components also have five times more ligand-receptor interactions than between GSC and OUT. The GSC component predominantly features 'transcriptional regulation' interactions, which appear ten times more often in the GSC than the OUT, and six times more often than the IN components. The OUT component is enriched with 'incoming' interactions such as transcription factor target. Indeed, 48% of interactions between the GSC and OUT are transcription regulation compared with 4% of interaction between IN and GSC being transcription regulation. The GSC encompassed around 50% of the HK network. Overall, the fraction of HK genes was larger in the GSC (*p *< 10-5) and slightly lower in the IN and OUT component than a random gene set of the same size (Figure 4C). This suggests that HK proteins comprise a significant proportion of signal transduction interactions. Mammary gland and tonsil were enriched in GSC (*p *< 0.08 and *p *< 0.14); intestine and adult kidney in OUT component (*p *< 0.03 and *p *< 0.07), and skin (*p *< 0.1) in IN component (Figure 4C, Additional file 12).

Next, all HK and tissue-specific sets were divided into protein classes according to the MetaCore protein classification schema (Additional file 13). Enrichment in proteins of a certain class was then calculated from the hypergeometric distribution. The distribution of protein classes in the global interactome was used as a reference (Methods). HK proteins were enriched in enzymes (*p *< 10-8). Intestine, liver and adrenal gland featured the highest fraction of enzymes. Fetal tissues were highly enriched in transcription factors, and retina and brain in membrane receptors (Figure 4B). Such distributions make intuitive biological sense, and both network structure and protein class enrichment were consistent with each other.

### Tissue-specific networks

**Figure 5:**
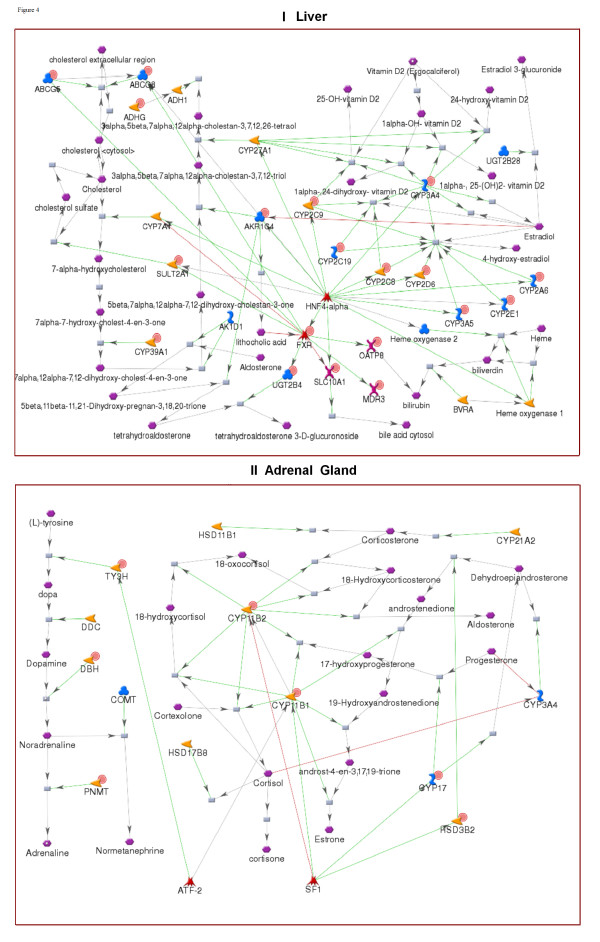
**Tissue-specific networks**. (I) Liver; (II) Adrenal gland. Genes identified as tissue-specific are marked with solid red circles.

We generated tissue-specific networks for nine tissues using the Analyze Networks (AN) network algorithm in MetaCore. AN is a version of the 'shortest path' algorithm, optimized for larger data sets. AN generates overlapping sub-networks of up to 50 nodes and calculates enrichment of the networks with input data and canonical pathways [17,18]. Unlike pre-built GG process networks, AN networks are built from input lists of network objects using the manually curated interaction database in MetaCore. The networks comprise metabolic reactions, signaling interactions and canonical pathways. The tissue-specific networks for liver and adrenal gland are shown in Figure 5. The liver network is enriched with cholesterol metabolism, as well as with enzymes involved in aldosterone, estradiol and heme metabolism. The adrenal network reflects the hormonal function of that organ. It is comprised of reactions and enzymes taking part in adrenaline, noradrenaline and corticosteroid synthesis.

### Clustering tissues based on gene expression patterns

**Figure 6:**
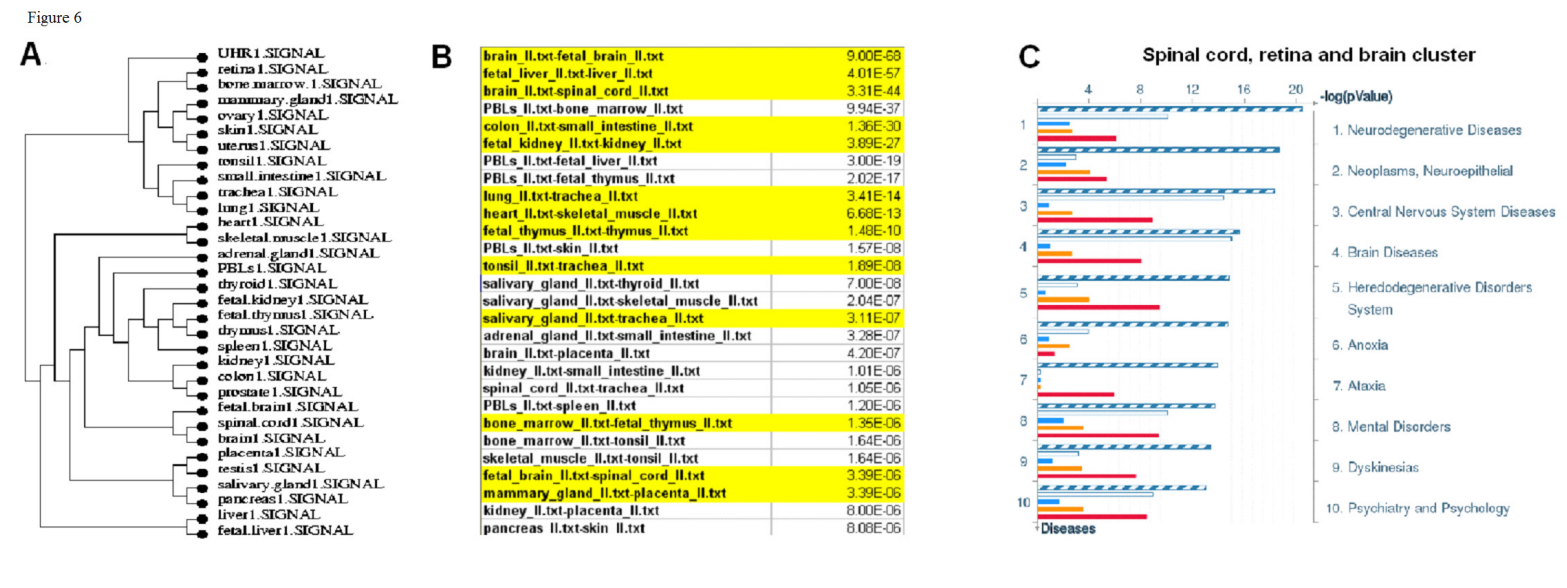
**Clustering tissues into groups based on expression patterns**. (a) Hierarchical clustering of genes based on gene expression data. (b) Tissue pairs having a significantly large number of genes uniquely expressed in them. (c) Proteins uniquely expressed in spinal cord, retina and brain are involved in diseases of the nervous system.

Tissue gene expression patterns were clustered by Euclidean distance across the average normalized probe intensities of the replicate hybridizations for each tissue [18] (Figure 6A). Most tissue clusters display evolutionary and functional relatedness. Fetal tissues clustered closest to their adult counterparts (brain-fetal brain, liver-fetal liver, thymus-fetal thymus, Figure 6A). Tissues of the same developmental origin (ectodermal, mesodermal or endodermal) tended to cluster together; for instance, heart and skeletal muscle are both derived from mesoderm, pancreas and salivary gland are part of the gastrointestinal system. Almost all of the most closely correlated tissue pairs were evolutionary or functionally related. Some genes were uniquely expressed in pairs of tissue (Figure 6B), rather than individual tissues (for instance, in brain and fetal brain) or in triplets and quadruplets of tissues (brain, fetal brain and spinal cord) (Additional file 14). Often, these proteins were specific for a group of diseases. For instance, proteins uniquely present in spinal cord, retina and brain are involved in central nervous system and neurodegenerative diseases (Figure 6C).

### Drug target distribution between housekeeping and tissue-specific genes

We compared the distribution of drug targets between HK and tissue-specific protein sets. Two sets of drug targets were compiled: 'therapeutic targets' – a set of 104 direct protein targets of commercially available drugs, and 'xenobiotic targets' – 518 proteins known to interact with xenobiotics. Therapeutic drugs are defined as key proteins or genes directly affected by a marketed or withdrawn drug from the market. Xenobiotic targets are proteins and genes known for physical interactions with a large set of bioactive compounds including drugs, drug candidates and lead compounds.

On average, therapeutic targets were twice as prevalent among tissue-specific proteins than HK proteins (3.3% and 1.5% of all proteins, correspondingly) (Additional file 15). Therapeutic targets comprised as much as 25% of mammary gland and thymus-specific proteins. The distribution of xenobiotic targets was not essentially different between HK and the sum of tissue-specific proteins, with some 1.7% of HK proteins and 2.3% of tissue-specific proteins being xenobiotic targets. Some tissues, however, were highly enriched with xenobiotic targets. For instance, over 10% of proteins specific for mammary gland, ovary, retina, small intestine and spinal cord were identified as xenobiotic targets (Additional file 15).

## Discussion

The definition of a 'housekeeping' or 'universally-expressed' gene is not settled. Suggested approaches vary from an estimated 10% of all genes [4] to transcript copy number [3] to using 'present' calls [5]. Elisenberg and Levanon [2] assumed that HK genes are highly expressed 'by nature' and, therefore, must be selected for shorter introns. We followed the original 'common-sense' proposal of Watson et al. [1] to define 2374 genes as HK because their transcripts were detected in all 31 human tissues tested, at a signal-to-noise ratio ≥ 10, which we believe to be a relatively stringent cut-off (technical analysis of platform sensitivity at ABI has indicated that a signal-to-noise ratio of ≥ 3 is sufficient to determine 'presence' of a transcript with a confidence of 99.9%. This set constitutes 8.2% of human genes based on the latest National Center for Biotechnology Information genome release .

Tissue-specific gene sets, comprising genes uniquely expressed in individual tissues by the SN10 criterion, averaged 43 genes for somatic tissues, and 485 mostly meiosis-specific genes in testis. The tissue specificity distribution is represented by a bimodal distribution with two peaks at 1 and 31 corresponding to tissue-specific and HK genes (Additional file 16). Interestingly, the female reproductive organ, ovary, expressed many fewer unique genes, probably due to the fact that ovary consists mainly of diploid tissue compared with the haploid-rich testis, as ovary produces far fewer eggs than testis does sperm.

There is continuing debate in gene expression analysis over how to best identify genes differentially expressed between treatment conditions, disease states and so on. Arbitrarily applied cut-offs in relative magnitude of expression (fold-change) and statistical measures such as ANOVA and *t*-test are commonly used [19,20]. Renewed debate was sparked recently [21,22] following publication of the results of the Microarray Quality Control Consortium research efforts [23]. It was shown that a combination of non-stringent statistical cut-offs and fold-change ranking of gene lists resulted in improved concordance between measurements made using different expression analysis technologies, an approach likely therefore to give the most accurate biological 'answer'.

We applied both signal level (signal-to-noise) and statistical (*t*-test) methods to determine tissue specificity of gene expression. Although the resulting datasets did not overlap well in gene content (6% on average), they were highly similar by functional analysis. Importantly, the intersections between distributions of entities within functional ontologies for SN10 and *t*-test sets were substantially larger than between genes (Additional file 3), suggesting that the two methods both select biologically relevant genes, which can be interconnected on pathways and networks. Further, despite incomplete overlap between our HK set and four other previously published HK sets, all gave very similar results in ontological enrichment analyses. These observations support the use of functional descriptors (pathways, networks, ontological categories and so on) as tools for quantitative characterization of conditional gene expression [24].

HK and tissue-specific gene sets were subjected to comprehensive functional analysis in three steps: enrichment analysis across functional ontologies using hypergeometric distribution [12] and GSEA methods [13]; local interactome analysis; generation of signaling/metabolic networks using HK and tissue-specific gene content as input nodes. We used multiple functional ontologies for enrichment analysis: GO processes, GG processes, disease categories and canonical pathway maps. Each ontology is established using different criteria and reveals a different aspect of cellular functionality. Canonical pathways are experimentally confirmed multi-step chains of reactions. GO and GG processes differ, as connectivity is established at the level of functional association (GO processes) or binary interactions (GG processes). Diseases and disease network ontologies represent pathways involved in pathological processes, largely derived from literature data linking network objects (genes, proteins, metabolites) to disease.

Ontology enrichment showed that HK genes, indeed, were enriched in vital cellular functions such as oxidative phosphorylation, ubiquitin-proteasomal proteolysis, translation and major pathways of endogenous metabolism. Importantly, many essential processes involve a large number of interconnected proteins (typically several hundred), and many of these genes belong to our HK set, confirming the functional integrity and completeness of the HK set.

Recently it was shown in line with our results that the number of HK genes is larger than previously reported [4,25]. Our findings are complementary to the high-resolution study based on expressed sequence tag (EST) profiling reported by Zhu et al. [25]. Indeed, both methods have advantages and drawbacks, EST data although have potentially higher resolution, suffers from poor annotation and low sampling depth [25]. Our results are very consistent throughout the 31 tissues and are well confirmed by enrichment analysis in multiple functional ontologies. We expect that higher resolution data would not alter the main conclusions of the enrichment and network analysis.

Ontology enrichment in tissue-specific genes was strikingly different from HK genes, and in most cases strikingly consistent with the tissue's function. Retina genes were enriched with such processes as visual perception, detection of visible light, detection of light stimulus and eye disease-related genes, and testis genes scored highest for reproductive processes, cell cycle, cell division and male reproductive diseases, for example. Importantly, almost all tissues featured a high level of consistency between the highest-scored categories in different ontologies. Further, consistency was observed whether hypergeometric *p*-values or GSEA analyses were employed. This important observation adds confidence to the conclusions drawn from the analysis of tissue-specific gene expression, and can be applied similarly to other studies of differentially expressed genes and proteins. It also will facilitate the development of a comprehensive set of 'meta-ontologies' derived from an understanding of the interplay and crossover between the different types of ontological categories available, leading to improved understanding of biological systems, and the identification of biologically meaningful biomarkers and new therapeutic targets.

Network analysis [15] showed that a large part of HK genes belonged to the most interconnected GSC core of the global interactome, with equal representation of 'in' and 'out' interactions. On the other hand, tissue-specific genes varied greatly in network component composition. Skin and fetal brain were enriched with 'IN' component, consistent with a larger proportion of ligand-receptor interactions in these tissues. Not surprisingly, these tissues featured a large fraction of receptors in the protein class enrichment test. In comparison, 'effector' organs such as intestine, liver and kidney, were enriched with 'OUT' interactions and enzymes. A transcription factor-enzyme pair represents a typical directed 'out' interaction – a final step in delivering a signal from stimulus to core 'effectors' such as endogenous metabolic pathways. These findings are in line with the 'bow-tie' structure of metabolic networks, which ensures robustness of major biological functions [26], and is needed for coordinated response to stimuli and perturbation [27].

'Multi-dimensional' functional analysis of gene expression data adds to the ongoing debate on proper statistical procedures for gene set selection. A 'rule of thumb' opinion is that only gene sets and distributions with low *p*-values (typically, *p *< 0.01) should be considered valid, although such *ad hoc *cut-offs may eliminate many condition-relevant genes from analysis. Functional analysis provides a different level of validation for such datasets by consideration of functional biological units. For instance, the retina-specific gene set is enriched in vision-related pathways, GO and GG ontologies, and diseases. The same set is enriched with OUT network component, and the protein class 'receptors'. Self-assembling retina-specific networks reconstruct rhodopsin-stimulated signal transduction and key steps in the metabolism of rhodopsin and its co-factors. Although the retina gene set is too small to achieve *p*-values < 0.01 in most of these analyses, the combination of independent functional evidence builds a strong case for its relevance and importance as a highly likely source of specific biomarkers for eye conditions and ophthalmic drug response. The comprehensive biological interaction data in MetaCore allows multiple 'high-content' data types to be mapped onto networks. Gene expression data, proteomic, single nucleotide polymorphism and metabolomic data all can be addressed, visualized and used in network construction and analysis, independently or in concert [28], making it a universal tool for studies on eye and other diseases.

Tissue-specific gene sets (and, therefore, networks) were twice as enriched in drug targets as the HK set. 'Therapeutic' targets comprised up to 25% of mammary gland and thymus-specific proteins. Most of the identified tissue-specific drug target genes are targets to drugs whose mechanism are consistent with the tissues. For example, three brain-specific proteins identified to have at least one drug target are GABA-A receptor beta-2 subunit, Na (V) I alpha and SCN10A. GABA-A receptor beta-2 subunit is a target for clomethiazole (sedative and anticonvulsant), Na (V) I alpha is a target for drugs such as levetiracetam (epilepsy), tetrodotoxin (anesthetics), toprimate (anticonsulvant) and SCN10A is target for bupivacaine racemic (anesthetics) and lidocaine (anesthetics).

Tissue-specific proteins and their pathways and networks are therefore a potentially rich source of targets and biomarkers for disease treatment and diagnostics. The addition of data on which network components are detectable in blood and other readily accessible body liquids makes tissue-specific networks exceptionally attractive for the identification of putative biomarkers of tissue-specific disease, drug-response or toxicity.

## Methods

### Data description

RNA from 31 normal human tissue RNAs (Table 1) was purchased from Clontech (Palo Alto, CA) while RNA from UHR (Universal Human Reference) was purchased from Strategene (San Diego, CA). All RNA samples were analyzed on the Agilent 2100 Bioanalyzer for RNA quality control. Three technical replicates were included for each of the tissue sample.

The Applied Biosystems Human Genome Survey Microarray (P/N 4337467) contains 31,700 60-mer oligonucleotide probes representing 27,868 individual human genes. Digoxigenin-UTP labeled cRNA was generated and amplified from 1 μg of total RNA from each sample using Applied Biosystems Chemiluminescent RT-IVT Labeling Kit v 1. 0 (P/N 4340472) according to the manufacturer's protocol (P/N 4339629). Fifteen micrograms of DIG-labeled cRNA was hybridized for 16 hrs at 55°C and chemiluminescence detection, image acquisition and analysis were performed using Applied Biosystems Chemiluminescence Detection Kit (P/N 4342142) and Applied Biosystems 1700 Chemiluminescent Microarray Analyzer (P/N 4338036) following the manufacturer's protocol (P/N 4339629). Images were auto-gridded and the chemiluminescent signals were quantified, background subtracted, and finally, spot- and spatially-normalized using the Applied Biosystems 1700 Chemiluminescent Microarray Analyzer software v 1. 1 (P/N 4336391). Probe signals were normalized using the Limma method [29]. The gene expression data is publicly available at GEO public repository website. (GSE7905).

### Identification of HK and tissue-specific genes

A 'stringent' set of HK genes expressed in each of the 31 tissues was identified by applying cut-off to the ABI-calculated signal-to-noise (S/N) for each probe. The S/N is a metric that captures the confidence of the measurement 'detectability' above all known sources of noise. S/N is commonly used to bin genes or probes as 'Present' or 'Absent' at a desired level of confidence. Since the S/N expresses the number of standard deviations, the associated confidence can be looked up from a probability table for a normal distribution. For example, signals with S/N ≥ 3 have > 99.9% confidence in the measurement. For our purposes in determining whether a gene was expressed in a given tissue, we additionally took into account the inflection points where the slope of the S/N curve significantly changes. We applied the threshold across all three replicate hybridizations on a probe-by-probe basis to identify the genes expressed in each tissue with a high level of confidence. The overlap between all 31 sets, that is, genes consistently expressed in all tissues, defines the HK set (more details in Additional file 17). Tissue-specific genes were defined as those uniquely expressed in a single given tissue at a S/N ≥ 10. Genes expressed in highly related tissue pairs were also defined as tissue specific for some analyses.

### Topological measures

#### Degree of nodes

The number of links connected to a node gives the node's degree. Since many real networks are directed, nodes are characterized by in and out-degree, giving the number of incoming and outgoing interactions.

#### Average shortest path

The shortest distance between two nodes is the number of links along the shortest path. The average shortest path is the average over the shortest paths for all node pairs in the network. When we calculate the shortest paths for a subset of nodes in the network we consider also paths crossing through nodes that are not part of the subset.

#### Average clustering coefficient

The clustering coefficient is a measure that captures to what degree node's neighbors are connected. It is defined as:

$$C_{i}=\frac{2n_{i}}{k_{i}(k_{i}-1)}$$

where *n*_*i *_is the number of links among the *k*_*i *_neighbors of node *i*. As *k*_*i*_*(k*_*i*_*-1)/2 *is the maximum number of such links, the clustering coefficient is a number between 0 and 1. The average clustering coefficient is obtained by averaging over the clustering coefficient of individual nodes. A network with high clustering coefficient is characterized by highly connected sub-graphs.

### *P*-value calculation

The enrichment levels of the HK genes and tissue-specific genes in the different parts of the network (IN, OUT, GSC) and in different protein classes were calculated using the hyper-geometric distribution:

$$P(k,D,n,N)=\frac{\left(\begin{array}{c} D\\ k \end{array}\right)\left(\begin{array}{c} N-D\\ n-k \end{array}\right)}{\left(\begin{array}{c} N\\ n \end{array}\right)}$$

We calculated the *p*-value corresponding to the enrichment-level according to

$$p(k)=\sum_{i=k}^{D}P(i,D,n,N)$$

which gives the probability of having *k *or more marked elements in a sample of size *n *by random selection.

## Competing interests

The authors declare that they have no competing interests.

## Authors' contributions

TN, YN and ZD conceived the study and designed research. ZD drafted and YN wrote the final manuscript. RJB contributed to writing and editing of the manuscript. ZD, WS and ES performed the statistical analysis of the data. TS, DD, AB, AG, ES, ZD and YN performed research. ER contributed with making the figures in the manuscript. KL, JB, RRS designed and carried out the experiment

## Supplementary Material

Additional file 1 **List of all housekeeping genes** Click here for file

Additional file 2 **List of all tissue-specific genes** Click here for file

Additional file 3 **Table for intersection between genes, ontologies** Click here for file

Additional file 4 **Complete enrichment analysis for all housekeeping sets** Click here for file

Additional file 5 **Canonical pathway maps and GeneGo networks essential for growth and viability** Click here for file

Additional file 6 **Enrichment analysis of the unique parts of the housekeeping gene sets** Click here for file

Additional file 7 **Complete enrichment analysis of all tissues in all four ontologies** Click here for file

Additional file 8 **Gene Set Enrichment Analysis for selected tissues, ontologies** Click here for file

Additional file 9 **Network topological properties of all housekeeping sets** Click here for file

Additional file 10 **Tissue-specific connectivity** Click here for file

Additional file 11 **Table for interactions mechanisms** Click here for file

Additional file 12 **Table for component analysis, protein class analysis for all tissues** Click here for file

Additional file 13 **MC legend with protein classes** Click here for file

Additional file 14 **List of genes specific for tissue pairs and triplets** Click here for file

Additional file 15 **Drug targets** Click here for file

Additional file 16 **Tissue-specificity distribution of all genes** Click here for file

Additional file 17 **Definition of tissue specific and housekeeping genes** Click here for file
