# Supplementary material for: A comprehensive functional analysis of tissue specificity of human gene expression
Source: BMC Biol. 2008 Nov 12;6:49. doi: 10.1186/1741-7007-6-49 (PMC2645369; doi:10.1186/1741-7007-6-49)
Supplement: Additional file 6 — Enrichment analysis of the unique parts of the housekeeping gene sets [file 1741-7007-6-49-S6.doc]

| **Enrichment by** | **Unique HK_genes**  **(threshold 10)** | **Unique for Tu data set** | **Unique for Tsyganskaya data set** | **Unique for Levanon data set** |
| --- | --- | --- | --- | --- |
| **Canonical pathways maps** | Ligand-dependent transcription of retinoid-target genes | Spindle assembly and chromosome separation | Oxidative phosphorylation | Antigen presentation by MHC class I |
|  | GTP-XTP metabolism | IGF-RI signaling | Cytoskeleton remodeling | Mitochondrial ketone bodies biosynthesis and metabolism |
|  | TCA | Antigen presentation by MHC class I | Integrin-madiated cell adhesion | Phenylalanine metabolism |
|  | CDC42 in cellular processes | MIF-JAB1 signaling | ECM remodelling | Neurophilaments |
|  | Formation of Sin3A and NuRD complexes and their role in transcription regulation | IL4 signaling pathway | Plasminogen biosynthesis | Transcription regulation of granulocyte development |
|  | CTP/UTP metabolism | CREB pathway | Mitochondrial unsaturated fatty acid beta-oxidation | Propionate metabolism p.2 |
|  | ATP/ITP metabolism | PIP3 signaling in cardiac myocytes | Regulation of actin cytoskeleton by Rho GTPases | Ligand-dependent transcription of retinoid-target genes |
|  | TGF, WNT and cytoskeletal remodeling | Cross-talk VEGF and angiopoietin1 signaling | Glucocorticoid receptor signaling | Tyrosine metabolism |
|  | Cadherin-mediated cell adhesion | IP3 signaling | Mitochondrial long chain fatty acid beta-oxidation | Receptor-mediated axon growth repulson |
|  | Role SCF complex in cell cycle regulation | Role of Akt in hypoxia induced HIF1 activation | Endothelial cell contacts by non-junctional mechanisms |  |
| **GeneGo processes** | Transcription_Nuclear receptors transcriptional regulation | Transcription_Chromatin modification | Muscle contraction | Immune_Phagosome in antigen presentation |
|  | Transcription_Transcription by RNA polymerase II | Inflammation_IL-12.15.18 signaling | Cell adhesion_Platelet aggregation | Reproduction_GnRH signaling pathway |
|  | Translation_Translation in mitochondria | Transcription_Nuclear receptors transcriptional regulation | Protein folding_Folding in normal condition | Immune_Antigen presentation |
|  | Proteolysis_Ubiquitin-proteasomal proteolysis | Inflammation_IL-6 signaling | Cell cycle_G2-M | Transmission of nerve impulse |
|  | Transcription_Chromatin modification | Inflammation_Amphoterin signaling | Cell adhesion_Integrin-mediated cell-matrix adhesion | Signal transduction_ESR1-nuclear pathway |
|  | Protein folding_ER and cytoplasm | Cytoskeleton_Regulation of cytoskeleton rearrangement | Protein folding_Protein folding nucleus | Cytoskeleton_Regulation of cytoskeleton rearrangement |
|  | Cell cycle_Mitosis | Cell cycle_Mitosis | Transcription_Transcription by RNA polymerase II | Cytoskeleton_Cytoplasmic microtubules |
|  | Cell cycle_G1-S | Inflammation_Inflammasome | Development_Heart development | Development_Regulation of angiogenesis |
|  | Protein folding_Folding in normal condition | Cytoskeleton_Actin filaments | Development_Cartilage development | Transcription_Transcription by RNA polymerase II |
|  | Cell cycle_S phase | Inflammation_IL-2 signaling | Reproduction_Feeding and Neurohormones signaling | Cytoskeleton_Intermediate filaments |
| **GO processes** | Macromolecule metabolic process | Organelle organization and biogenesis | Inositol phosphate-mediated signaling | Negative regulation of synaptic transmission |
|  | Primary metabolic process | Cellular component organization and biogenesis | Ventricular system development | Negative regulation of transmission of nerve impulse |
|  | Cellular metabolic process | Establishment of cellular localization | Cardiac muscle cell development | Gamma-aminobutyric acid secretion |
|  | Metabolic process | Negative regulation of interleukin-12 biosynthetic process | Cardiac myofibril assembly | Negative regulation of nucleotide metabolic process |
|  | Biopolymer metabolic process | Cellular localization | Third ventricular development | Protein retention in ER |
|  | Nucleobase, nucleoside, nucleotide amd nucleic acid metabolic process | Intracellular transport | Lateral ventricular development | Protein localization |
|  | Leading adge cell differentiation | Antibacterial humoral response (sense Vertebrata) | Fourth ventricle development | Negative regulation of neurological process |
|  | Cell cycle | Intracellular protein transport | Serotonin receptor signaling pathway | Receptor clustering |
|  | Positive regulation of transcroption | Antibacterial humoral response | Serotonin receptor, phospholipase C activating pathway | Intracellular protein transport |
|  | Transcription from RNA polymerase II promoter | Protein transport | Adult heart development | Synaptic transmission, glutamatergic |
| **Diseases** | Papilloma | Hyaline membrane disease | Classic Migraine | Sleep disorders, Circadian rhythm |
|  | Breast neoplasms | Respiratory distress syndrome, newborn | Emotions | Chronobiology disorders |
|  | Breast diseases | Incontinentia pigmenti | Anxiety | Delirium |
|  | Adenocarcinoma | Multiple trauma | Disorders of environmental origin | Medulloblastoma |
|  | Leukemia, Monocytic< Acute | DNA damage | Heart diseases | Confusion |
|  | Skin diseases | Epiretinal membrane | Depressive disorder | Occulopational diseases |
|  | Neoplasms by site | Spondylitis | Colonic diseases, functional | Alcohol withdrawl delirium |
|  | Neoplasms, glandular and epithelial | Spondylitis, Ankylosing | Irritable Bowel syndrome | Pregnancy, Tubal |
|  | Endocrine gland neoplasms | Ankylosis | Depressive disorder, Major | Cluster headache |
|  | Lung neoplasms | Bone diseases, Infectious | Aneurysm | Alcohol-induced disorders, Nervous system |
