# Supplementary material for: A comprehensive functional analysis of tissue specificity of human gene expression
Source: BMC Biol. 2008 Nov 12;6:49. doi: 10.1186/1741-7007-6-49 (PMC2645369; doi:10.1186/1741-7007-6-49)
Supplement: Additional file 10 — Tissue-specific connectivity [file 1741-7007-6-49-S10.doc]

**(a)** **(b)**

| **Tissue** | **Average**  **path** | **Tissue** | **Degree** |
| --- | --- | --- | --- |
| Liver | 3.02 | Colon | 53.25 (p=0.015) |
| Ovary | 3.14 | Ovary | 21.14286 (p=0.06) |
| Adrenal_gland | 3.28 | Fetal_kidney | 21 |
| fetal_kidney | 3.33 | Placenta | 14.94444 |
| Thyroid | 3.33 | Bone_marrow | 12.19231 |
| Tonsil | 3.36 | Mammary_gland | 11.38462 |
| small_intestine | 3.39 | Spleen | 11.1 |
| Colon | 3.59 | fetal_thymus | 10.8 |
| Skin | 3.53 | skeletal_muscle | 10.6 |
| Mammary_gland | 3.53 | fetal_liver | 10.375 |
| Spleen | 3.55 | Skin | 9.47619 |
| fetal_liver | 3.60 | fetal_brain | 9.315789 |
| Placenta | 3.63 | Tonsil | 9.066667 |
| Kidney | 3.63 | adrenal_gland | 9 |
| Trachea | 3.64 | PBLs | 8.78125 |
| Heart | 3.65 | Liver | 8.142857 |
| Thymus | 3.66 | Lung | 8.090909 |
| Lung | 3.69 | Retina | 7.612069 |
| Skeletal_muscle | 3.69 | Thymus | 7.333333 |
| Prostate | 3.7 | Brain | 7.238095 |
| Uterus | 3.71 | Heart | 6.857143 |
| PBLs | 3.79 | Uterus | 6.571429 |
| fetal_thymus | 3.8 | Thyroid | 6.333333 |
| bone_marrow | 3.80 | Pancreas | 6.3 |
| fetal_brain | 3.84 | Trachea | 6.125 |
| Retina | 3.85 | Kidney | 6.083333 |
| Brain | 3.92 | spinal_cord | 6 |
| Pancreas | 3.93 | small_intestine | 6 |
| spinal_cord | 4 | Testis | 5.211538 |
| Testis | 4.17 | salivary_gland | 4.555556 |
| Salivary_gland | 4.22 | Prostate | 4 |

**
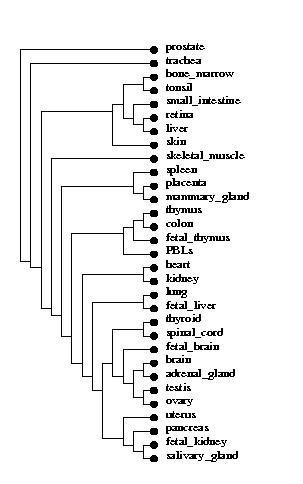
**

**Supplimentary figure 10. (a) Average shortest paths and connectivity of tissue specific proteins calculated in the global protein interaction network (b) Clustering of tissues based on network topological properties. The topological distance between two tissues was calculated as the average shortest path between node pairs corresponding to two different tissues divided by the average shortest path within the nodes of the same tissues.**
