# Supplementary figures and images for: A comprehensive functional analysis of tissue specificity of human gene expression
Source: BMC Biol. 2008 Nov 12;6:49. doi: 10.1186/1741-7007-6-49 (PMC2645369; doi:10.1186/1741-7007-6-49)

Protein classes in Metacore:


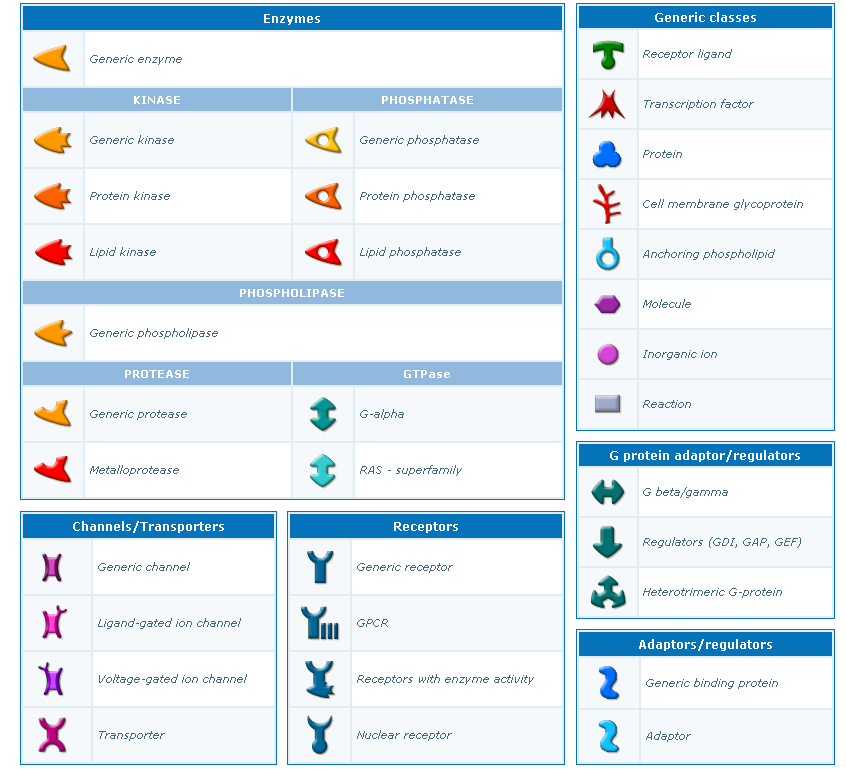

Supplement: Additional file 13 — MC legend with protein classes [file 1741-7007-6-49-S13.doc]
