# Supplementary material for: A comprehensive functional analysis of tissue specificity of human gene expression
Source: BMC Biol. 2008 Nov 12;6:49. doi: 10.1186/1741-7007-6-49 (PMC2645369; doi:10.1186/1741-7007-6-49)
Supplement: Additional file 16 — Tissue-specificity distribution of all genes [file 1741-7007-6-49-S16.docx]

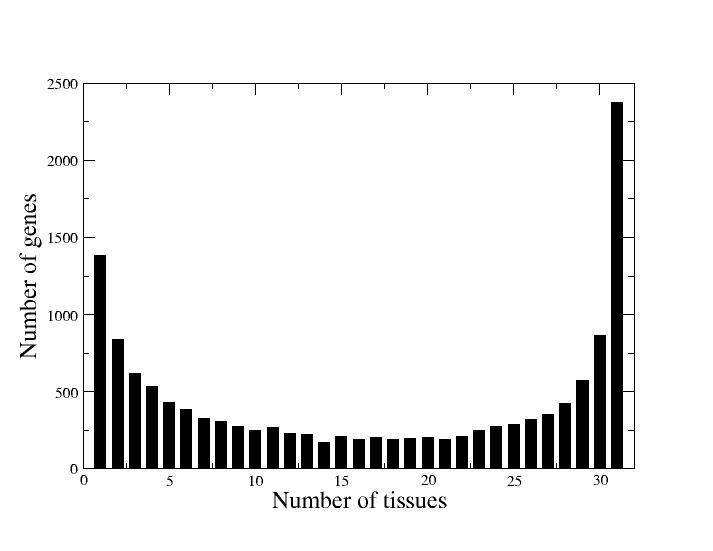


Tissue specific distribution of genes. The bimodal distribution shows to peaks at 1 and 31 corresponding to tissue specific and housekeeping genes.
