# Supplementary material for: A comprehensive functional analysis of tissue specificity of human gene expression
Source: BMC Biol. 2008 Nov 12;6:49. doi: 10.1186/1741-7007-6-49 (PMC2645369; doi:10.1186/1741-7007-6-49)
Supplement: Additional file 17 — Definition of tissue specific and housekeeping genes [file 1741-7007-6-49-S17.doc]

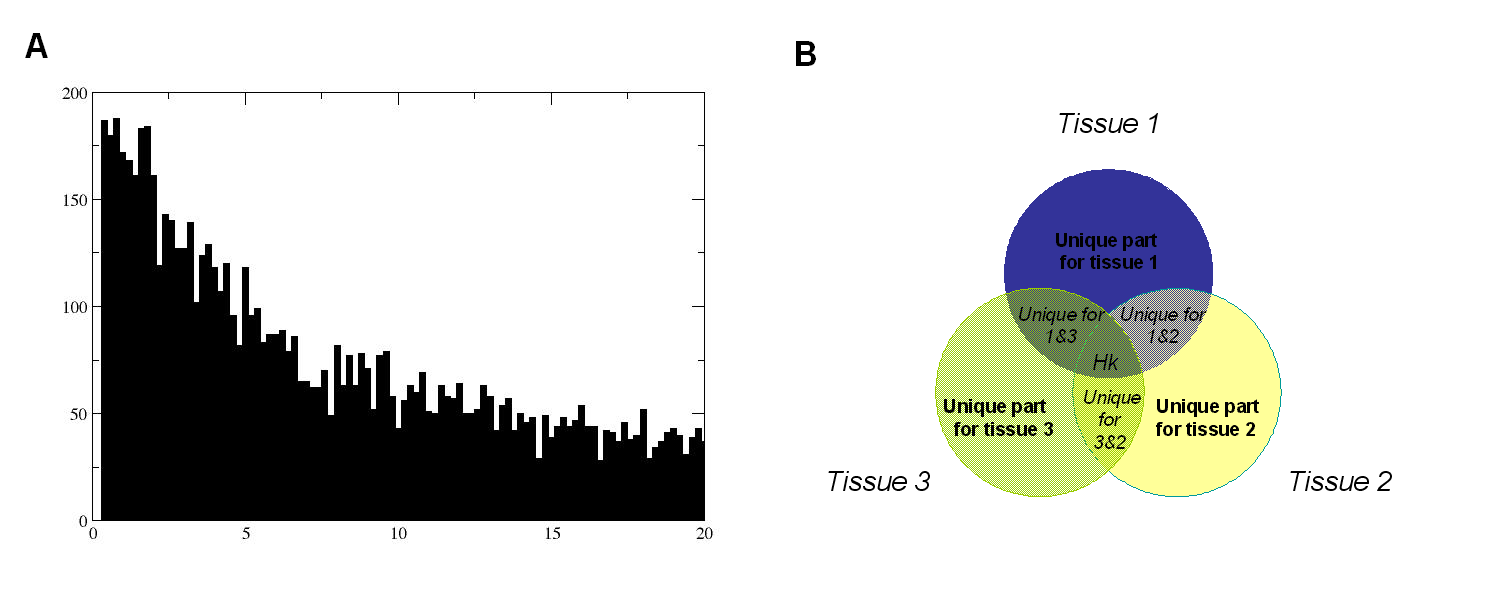


Definition of housekeeping and tissue specific genes

1. The S/N ratio distribution of retina, the inflection point being between 8 and 10 standard deviations
2. The intersection between sets of genes expressed for 3 hypothetical tissues after S/N>10 was applied. Different regions show the housekeeping, tissue specific (unique) and genes specific for tissue pairs.
